# Hydrophobic Fluorescent Probes Introduce Artifacts into Single Molecule Tracking Experiments Due to Non-Specific Binding

**DOI:** 10.1371/journal.pone.0074200

**Published:** 2013-09-16

**Authors:** Laura C. Zanetti-Domingues, Christopher J. Tynan, Daniel J. Rolfe, David T. Clarke, Marisa Martin-Fernandez

**Affiliations:** Central Laser Facility, Science & Technology Facilities Council, Research Complex at Harwell, Rutherford Appleton Laboratory, Didcot, United Kingdom; National Cancer Institute, United States of America

## Abstract

Single-molecule techniques are powerful tools to investigate the structure and dynamics of macromolecular complexes; however, data quality can suffer because of weak specific signal, background noise and dye bleaching and blinking. It is less well-known, but equally important, that non-specific binding of probe to substrates results in a large number of immobile fluorescent molecules, introducing significant artifacts in live cell experiments. Following from our previous work in which we investigated glass coating substrates and demonstrated that the main contribution to this non-specific probe adhesion comes from the dye, we carried out a systematic investigation of how different dye chemistries influence the behaviour of spectrally similar fluorescent probes. Single-molecule brightness, bleaching and probe mobility on the surface of live breast cancer cells cultured on a non-adhesive substrate were assessed for anti-EGFR affibody conjugates with 14 different dyes from 5 different manufacturers, belonging to 3 spectrally homogeneous bands (491 nm, 561 nm and 638 nm laser lines excitation). Our results indicate that, as well as influencing their photophysical properties, dye chemistry has a strong influence on the propensity of dye-protein conjugates to adhere non-specifically to the substrate. In particular, hydrophobicity has a strong influence on interactions with the substrate, with hydrophobic dyes showing much greater levels of binding. Crucially, high levels of non-specific substrate binding result in calculated diffusion coefficients significantly lower than the true values. We conclude that the physic-chemical properties of the dyes should be considered carefully when planning single-molecule experiments. Favourable dye characteristics such as photostability and brightness can be offset by the propensity of a conjugate for non-specific adhesion.

## Introduction

Single-molecule fluorescence imaging techniques are becoming popular tools for probing the structure and dynamic properties of living matter. Examples include the investigation of molecular localization and separation (NALMS [1], FIONA [2]), determining intermolecular distances in the range of 1–8 nm (FRET [3]) and 1–50 nm (FLIP [4]), sub-diffraction-limit imaging (PALM [5], STORM [6]), and determining the behaviour of mobile molecules either in solution or on the surface of live cells [7].

All single-molecule fluorescence techniques contend with the challenges of detecting weak signals above background noise and coping with fluorophore blinking and/or bleaching, which can limit the usefulness of a probe for a given single-molecule application. These issues make dye selection an important step in successfully planning a single-molecule experiment, particularly when using cell samples, which display increased background intensity due to autofluorescence. Photophysical characteristics such as quantum yield, high photostability, and resistance to blinking are normally the primary considerations when selecting dyes for single molecule experiments. However, we recently demonstrated that non-specific binding of organic fluorescent dye conjugates to the substrate on which cells are cultured can introduce non-trivial artifacts into single molecule tracking data acquired with total internal reflection fluorescence (TIRF) microscopes [8]. This is because dyes that are non-specifically bound to the substrate are immobile and will therefore skew the results of any analysis of molecular mobility in live cell experiments. Unfortunately, the problem cannot be resolved by simply ignoring immobile spots at the analysis stage, because molecules of interest can also be immobile [9].

Our previous systematic investigation focused on comparing the effectiveness of different methods for passivating the sample substrate to prevent non-specific binding of protein-dye conjugates. The predominant factor differentiating non-specific binding of fluorescent conjugates to the same substrate was shown to be the dye used. In this study we have compared a much larger range of dyes that can be excited with the commonly used 491 nm, 561 nm and 638 nm laser lines on a single substrate and conjugated to a single ligand of proven specificity, anti-EGFR Affibody [10].

Differences in chemical structure between dyes with similar spectral properties influence parameters such as brightness, photostability and electrostatic interactions, the latter influencing the propensity to adsorb onto a substrate. We investigated 14 different dyes from 5 different suppliers: Alexa 488, Alexa546, Alexa555, Tetramethylrhodamine (TMR), Bodipy FL, and Rhodamine Red C2 (Molecular Probes -Invitrogen), Cy3 (GE Healthcare), CF488A, CF568, CF633 and CF640R (Biotium), Atto565 and Atto647N (AttoTec), and Fluorescein (Sigma Aldrich), and determined for each its brightness, bleaching and non-specific adhesion when conjugated to the Affibody, the latter via its effect on the apparent diffusion of molecules on the basal membrane of live T47D cells grown on PEG-BSA nanogel passivated glass [11]. Maleimide derivatives of the dyes were used to label a single cysteine in the anti-EGFR affibody; in some cases, specific dye isomers were used and this is specified in the Materials and Methods. Our results demonstrate that spectrally similar dyes conjugated to the same protein can display large differences in non-specific binding and those desirable fluorescence properties of dye conjugates such as high brightness or photostability can be offset by their propensity for non-specific binding. High levels of binding to the substrate are associated with high levels of dye hydrophobicity.

## Results

### Overview of Dyes

Many different alternative dyes are available for every spectral class, each characterized by extinction coefficient, photostability, quantum yield, pH sensitivity and water solubility. Alexa Fluor and Atto dyes are commonly used for single-molecule applications, alongside cyanine dyes such as Cy3. Atto 647N is a popular dye in single-molecule experiments due to its outstanding brightness and photostability [12], however, this dye is positively charged and hydrophobic [13] and high levels of non-specific binding of Atto 647N conjugates have been reported previously [14]. CF-series dyes are a new class of dyes derived from the structures of coumarin, pyrene, rhodamine or cyanine [15], developed with the intention of improving water solubility, brightness and stability, as well as providing excellent specificity when conjugated to proteins and oligonucleotides [16], all characteristics which would be appealing for single-molecule work.

Information about the dyes used in this paper is summarised in **Table 1**. Net molecular charge ranges from strongly negative (Alexa Fluor 488) to moderately positive (Atto 647N). There is also a significant variation in the hydrophobicity of the dyes, as expressed by the log of the distribution coefficient, LogD, which is a measure of the expected ratio of dye concentrations in water and a non-polar solvent (octanol). LogD is defined as:

**Table 1:** Summary of dye characteristics.

Dye	Mean photon detection ratefrom a single molecule (s^−1^)	Apparent photobleachingtime constant (s)	net charge pH 7.4^1^	logD at pH 7.4^1^
**Alexa Fluor 488**	1164±181	15.1±0.2	−3.94	−10.48
**Bodipy FL**	2586±393	21.3±0.6	0	−1.99
**CF488**	1200±213	17.8±0.4	−3	−8.83
**Fluorescein**	2436±350	1.4±0.1	−1.9	−1.30
**Alexa Fluor 546**	2697±230	24.0±0.1	−3.41	−2.53
**Alexa Fluor 555**	1112±223	33.6±0.3	Unavailable	Unavailable
**Atto 565**	2850±535	14.5±0.2	0	−0.83
**CF568**	1042±194	40.7±0.5	−3	−3.74
**Cy3**	986±198	25.3±0.2	0	+3.03
**Rhodamine Red C2**	3268±453	8.3±0.1	−0.99	+1.53
**TMR6**	832±223	7.5±0.1	0	−5.6
**Atto 647N**	3290±231	36.0±0.2	+0.61	+1.96
**CF633**	851±170	16.4±0.1	−2	−5.44
**CF640R**	1084±202	37.7±0.2	−3	−10.29

1 Calculated from structures using “Marvin Sketch” software (Chemaxon). Structures of CF dyes are unavailable but charge and logD were calculated by the manufacturer, using the same method. Bold lines indicate divisions between groups of dyes excited at different wavelengths, as follows: Top four dyes, 491 nm; middle seven dyes, 561 nm, bottom three dyes, 638 nm. Laser flux exiting the objective was 3.2 µW/µm^2^ at 491 and 561 nm, 3.4 µW/µm^2^ at 638 nm.



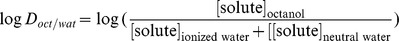



So a molecule with a negative value of logD (e.g. Alexa Fluor 488) is hydrophilic, and a molecule with a positive logD (Atto 647N) is hydrophobic.

### Assessment of Dye Brightness and Photostability for Single-molecule Experiments

Photostability and high brightness are essential characteristics for dyes used in single-molecule methods, in order to achieve the highest possible signal-to-noise ratios. First we tested the brightness and the photostability of the dye conjugates under the conditions we commonly use for 3 colour single molecule imaging. All dyes were used in their maleimide form and conjugated to an anti-EGFR Affibody molecule following manufacturer’s instructions. T47D cells were labelled with the affibody and imaged on a TIRF microscope. The mean number of photons emitted per single molecule and photobleaching time constants measured for each dye are also shown in **Table 1**. These measurements give a guide to how the dyes perform in our system for single molecule experiments, but will vary depending on the wavelength of illumination, laser power, choice of emission filter, and buffer conditions such as pH. Quantum yield and extinction coefficients also give a useful guide to a dye’s photophysical characteristics and expected performance; these can be obtained from the manufacturers.

For the dyes excited at 491 nm, Alexa 488 and CF488 produce similar numbers of photons per image frame, and photobleaching time constants show similar photostability. More photons are produced by Bodipy FL and Fluorescein, with Bodipy showing good photostability, unlike fluorescein, which, under the conditions used, has the shortest photobleaching time constant of all the dyes. Based on photophysical characteristics alone, Bodipy FL would appear to be the dye of choice for this wavelength range.

Of the 561 nm-excited dyes, Rhodamine Red C2, Atto 565, and Alexa 546 emit the highest numbers of photons per image frame. Of these dyes, Alexa 546 is the most photostable, with poor photostability being observed for Rhodamine Red C2. CF568, whilst emitting fewer photons, has the highest photostability of all the dyes in this wavelength range.

For 638 nm excitation, Atto 647N has a high number of emitted photons, and good photostability, and, based only on photophysical characteristics, would be the best dye excited at this wavelength. CF640R also shows good photostability, but produces fewer photons.

### Hydrophobicity is the Major Determinant for Non-specific Binding

Good photophysical characteristics are necessary but not sufficient for a dye to be suitable for single molecule imaging. A dye conjugate needs to be not only bright and stable, but also to be specific to its target so as to minimize artifacts in the data. We have previously demonstrated that non-specific binding of probes to the glass surface can introduce significant artifacts into data derived from single molecule images [8]. In our previous work we used mean instantaneous diffusion coefficient (*D*) values as a measure of the mobility of dye conjugates bound to receptors in the plasma membrane of T47D cells, and showed that anti-EGFR affibody conjugated to Alexa Fluor 488 exhibits similar levels of mobility when bound to EGFR in cells to endogenously labelled EGFR-GFP.

**Figure 1:**
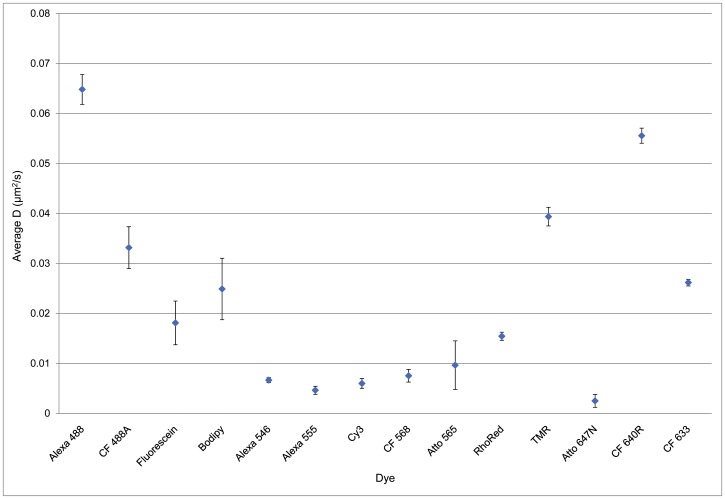
Mean instantaneous *D* fit for different anti-EGFR Affibody conjugates. Each datapoint corresponds to mean ± SEM of at least 10 areas acquired from 3 independent samples.

We have now investigated the mobility of a range of dye-EGFR affibody conjugates on T47D cells, using the value of *D* calculated for Alexa Fluor 488-labelled affibody as a reference. Mean *D* fit values calculated for all the dye conjugates are shown in **Fig. 1**. The figure indicates that all the other dye conjugates have lower mobility than Alexa Fluor 488. This was confirmed by a comparison with Alexa Fluor 488 using a Kolmogorov-Smirnov test. This test is non-parametric, requiring only that the distribution for each sample is continuous. All conjugates displayed significantly lower mobility (P≤0.001) than the reference, except CF640R (P = 0.097). The dyes excited at 561 nm performed particularly poorly, the best being TMR, which still shows only two thirds the mobility of the reference dye. Of the red dyes, CF 640R performs well, with a mobility close to that of the reference.

**Figure 2:**
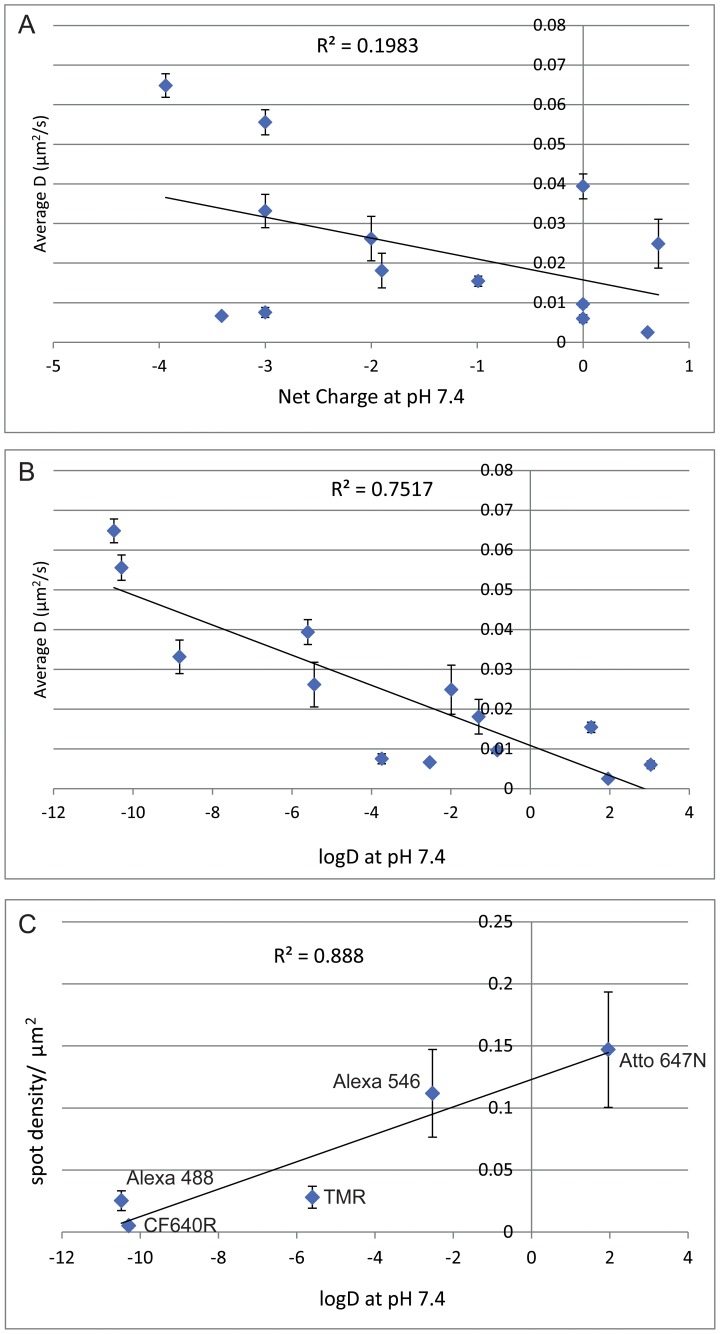
Effect of logD and charge on affibody conjugate mobility. Plots of mean instantaneous *D* fit for different anti-EGFR Affibody conjugates vs charge at pH 7.4 (**A**), and logD (**B**). **C)** Plot of spot density for selected anti-EGFR Affibody conjugates vs charge at logD. Each datapoint corresponds to mean ± SEM of at least 10 independent areas. Lines show linear regression fit to the data, R^2^ values indicating goodness of fit. Alexa 555 is not included in this figure as the structure is not published and charge and logD values are unavailable.

We then investigated the hypothesis that physicochemical properties of the dyes could be used to predict the degree of non-specific binding, and therefore be used to guide the selection of dyes for single molecule experiments. Two possible properties were investigated, net charge and hydrophobicity (logD). **Fig. 2** shows plots of diffusion coefficient vs net charge (**A**) and vs logD (**B**). Correlation between dye properties and diffusion coefficient was assessed by measuring the closeness of fit to a linear relationship. The data show a strong correlation between logD and dye conjugate mobility (R^2^ 0.75), but only a weak correlation between net charge and mobility (R^2^ 0.2). This indicates that dye hydrophobicity is a strong indicator of a dye’s propensity for non-specific binding. As an independent confirmation that dye hydrophobicity is correlated with non-specific binding, we also measured directly the density of conjugate binding to substrate for selected dyes. PEG-BSA nanogel treated glass substrates were exposed to dye conjugates and the number of fluorescent spots remaining after washing was counted. These data are plotted in **Fig. 2C**, which shows a strong correlation between logD and spot density, confirming the association between hydrophobicity and non-specific dye binding to the substrate.

**Figure 3:**
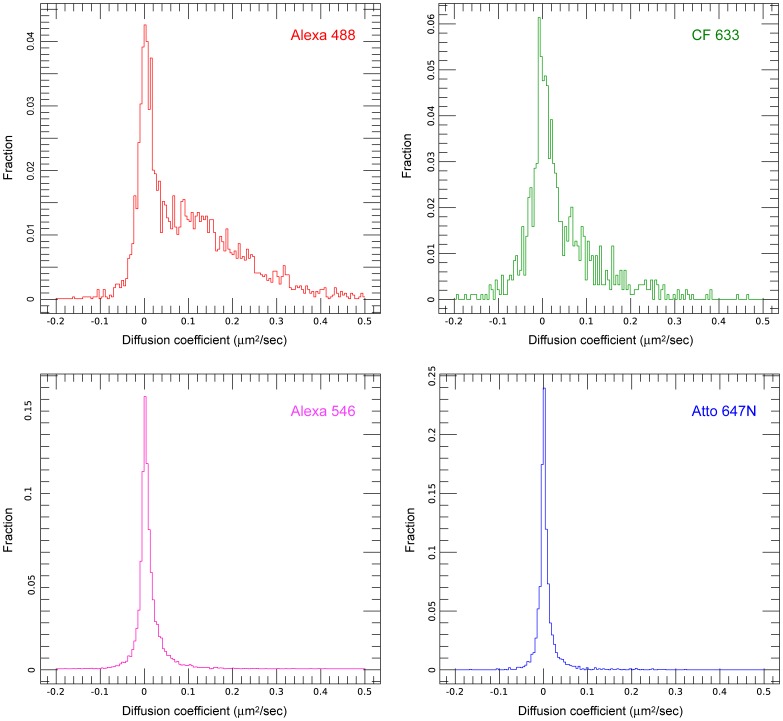
Plots of distributions of mean instantaneous *D* fits for affibody-dye conjugates. Dyes selected to represent high (Alexa 488), moderate (CF 633), low (Alexa 546), and very low (Atto 647N) spot mobility.

**Fig. 3** shows the distribution of *D* values for EGFR-GFP and affibody-dye conjugates selected for high, medium, low, and very low levels of mobility (Alexa Fluor 488, CF 633, Alexa Fluor 546, and Atto 647N, respectively). All *D* distributions show a peak at zero and a tail of varying magnitude extending out to >0.3 µm^2^/s. The zero peak will consist of a mixture of immobile molecules bound to the glass substrate, and immobile and slow-moving molecules in the cells, and the tail will correspond to mobile molecules in the cells. The less mobile dyes have a higher fraction of spots in the zero peak, consistent with higher levels of binding to the substrate. However, we also considered the possibility that the difference in mean diffusion coefficients measured for different dye conjugates was not only due to differences in levels of binding to the substrate, but also due to variations in the mobility of the conjugates when bound to EGFR in the plasma membrane. We investigated this by recalculating diffusion coefficients only including data from spots that were clearly mobile. In **Fig. 3**, negative *D* values can be observed, extending to approximately −0.1 µm^2^/s. As a spot cannot have a value of *D* below zero, we conclude that these negative *D* values are due to errors in D resulting mainly from localization errors, and that the maximum *D* error is of the order ±0.1 µm^2^/s. It is therefore a fair assumption to make that spots with values of *D* >0.1 µm^2^/s are definitely mobile, so we have taken this as the cutoff point for our analysis of only mobile spots.

**Figure 4:**
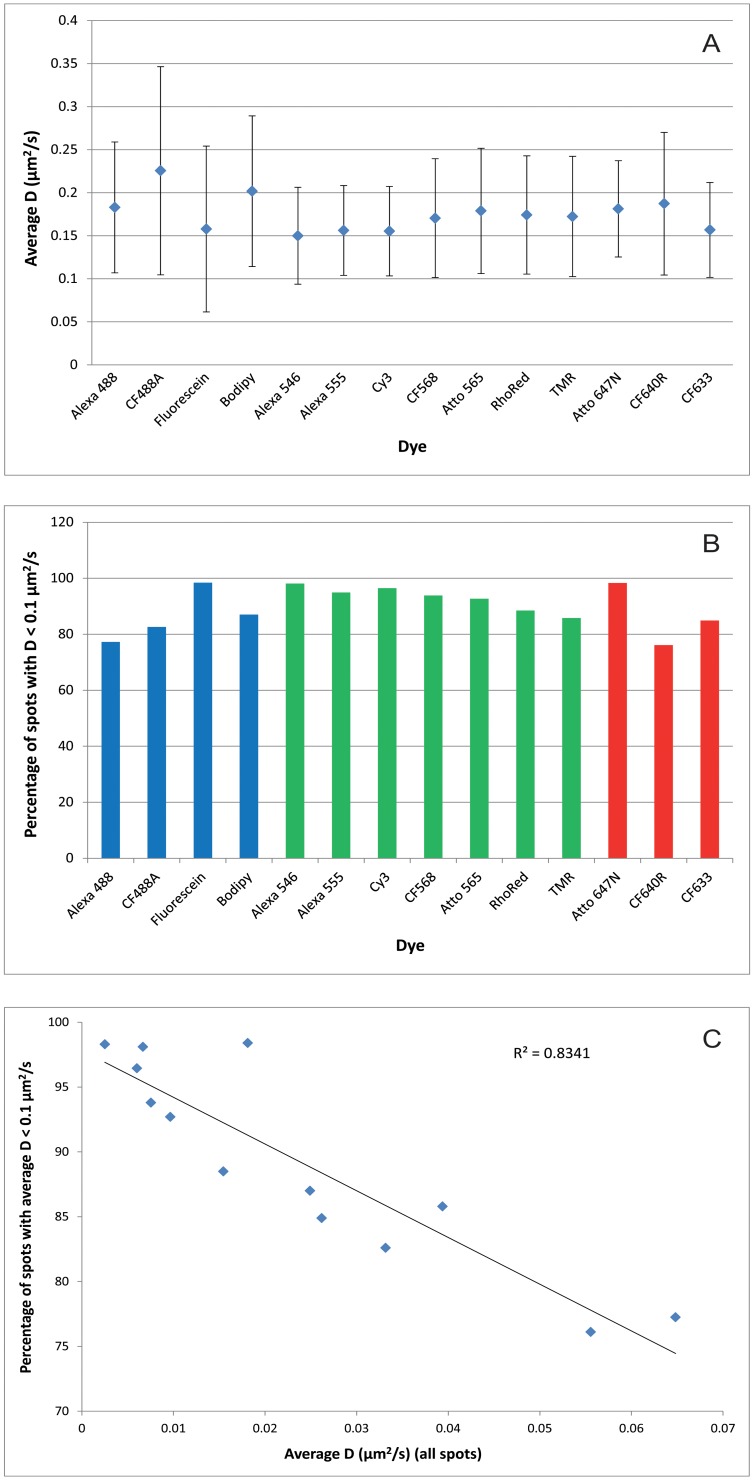
Analysis of definitely mobile vs immobile or very slow moving spots. **A)** Mean instantaneous *D* fit for different anti-EGFR Affibody conjugates, after removing data for spots with *D* values below 0.1 µm^2^/s. Each datapoint corresponds to mean ± SD of of the tracks contained in at least 10 different areas containing a minimum of 50 different cells. Blue bars indicate dyes excited at 491 nm, green at 561 nm, and red at 638 nm. **B)** Percentages of spots for each dye with *D* values below 0.1 µm^2^/s. **C)** Plot of mean instantaneous *D* fit for different anti-EGFR Affibody conjugates (calculated from all spots) vs percentage of spots with *D* values <0.1 µm^2^/s. Line shows linear regression fit to the data, R^2^ value indicating goodness of fit.

The results of the analysis of these “definitely mobile” spots are plotted in **Fig. 4A**, and show that we were unable to detect any significant differences in *D* values between the different conjugates. We have also plotted for each dye the percentage of spots with diffusion coefficients below the 0.1 µm^2^/s cutoff (**Fig. 4B**). Although all dyes show relatively high numbers of spots with measured diffusion coefficients below 0.1 µm^2^/s, dyes having the lowest mean diffusion coefficients have the lowest percentages of spots identified as definitely mobile. This is confirmed by plotting *D* values for all spots against percentages of spots falling below the 0.1 µm^2^/s cutoff (**Fig. 4C**). A linear fit shows a strong correlation (R2 = 0.83), with high levels of overall mobility being correlated with higher levels of unambiguously mobile spots. This adds weight to the argument that low diffusion coefficients largely result from higher levels of non-specific binding to the substrate.

**Figure 5:**
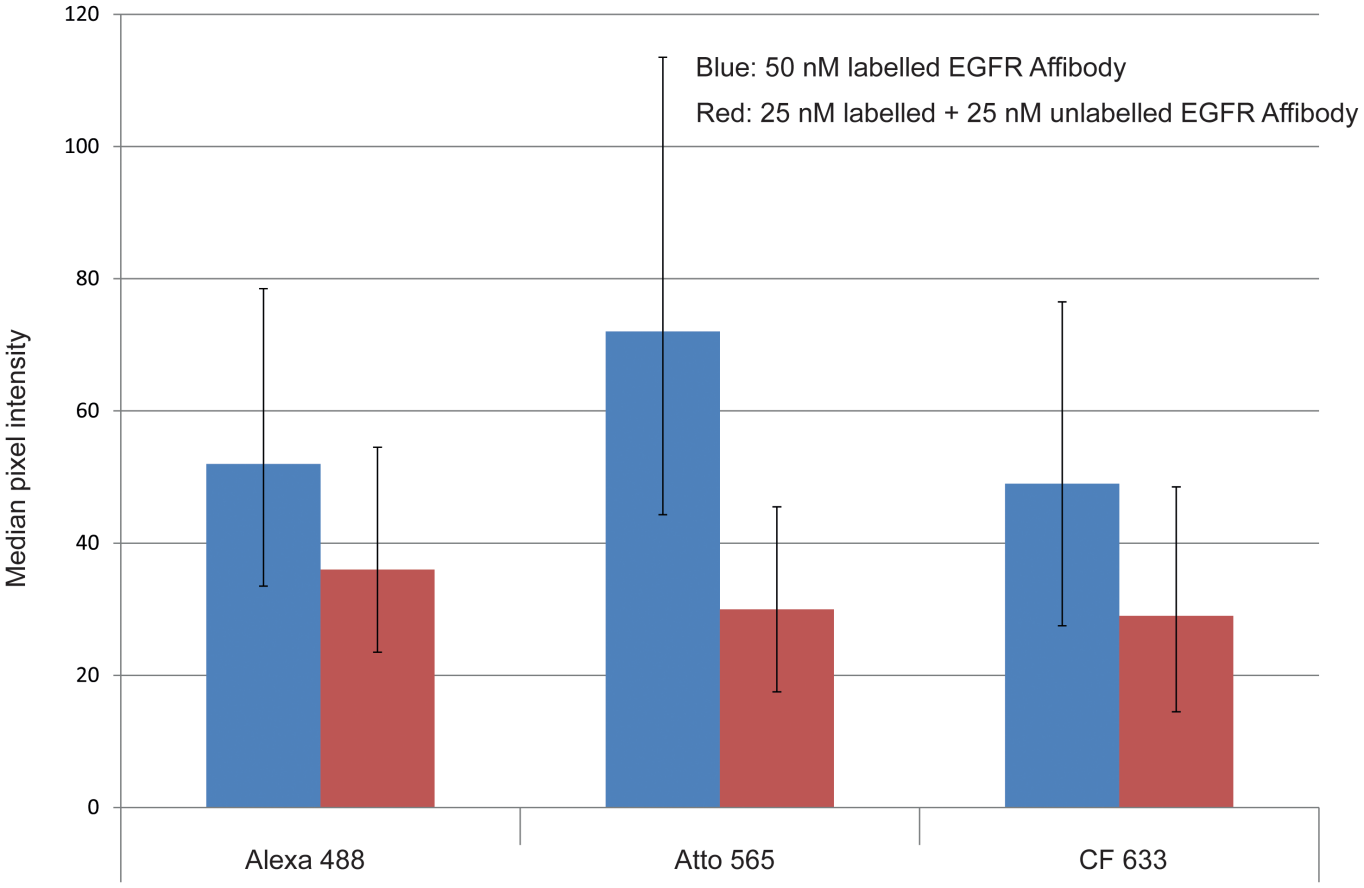
Fluorescence intensity measured from confocal microscopy images of T47D cells labeled with 50-conjugated EGFR affibody, and a mixture of 25 nM dye-conjugated affibody and 25 nM unlabeled affibody. Three dyes were selected to cover the range of mobilities (Alexa 488, high mobility; CF 633, moderate mobility; Atto 565, low mobility). Columns represent the median of the distribution of membrane region pixel intensities derived from at least 100 cells. Error bars represent the positions of the 1^st^ and 3^rd^ quartile of the distributions.

Finally, we considered the possibility that conjugation of dyes disrupts affibody function through unfolding, and that this effect may explain the variations in mobility that we have observed. To investigate this we have assessed the affinity of labeled affibody for its receptor, by measuring its degree of binding in competition with unlabeled affibody. These measurements were made for three affibody conjugates selected to cover the mobility range: Alexa 488 (high mobility), CF 633 (moderate mobility), and Atto 565 (low mobility). **Fig. 5** shows the fluorescence intensity measured from confocal microscopy images of T47D cells labeled with 50 nM dye-conjugated EGFR affibody, and a mixture of 25 nM dye-conjugated affibody and 25 nM unlabeled affibody. If the affinities of conjugated and unlabeled affibody are similar, we would expect the cells treated with the conjugated/unlabeled mixture to show approximately 50% of the fluorescence intensity of cells treated with only conjugated affibody. The data shown in **Fig. 5** are consistent with this. We observe no significant differences in the reduction of fluorescence intensity on addition of unlabeled affibody between the three affibody conjugates tested. In previously published work we have also shown that affibody-dye conjugates retain their specificity for EGFR, using competition assays with unlabeled affibody [8]. These data confirm that dye conjugation does not significantly reduce the affinity of the affibody for its target, and that the variations in conjugate mobility cannot be caused by varying levels of affibody unfolding on dye conjugation.

## Discussion

Single-molecule microscopy applications are uniquely able to obtain the distribution of values for different dynamic and structural parameters in a population of molecules, highlighting the molecular heterogeneity of the population based on the distribution of values, and allowing researchers to identify rare, transient states of a system and analyse dynamic processes without synchronization [17]. However, gaining a sufficient signal to noise ratio from organic fluorescent dyes can be demanding. As a result, when choosing dyes, it is tempting to make selections based exclusively upon consideration of photophysical properties, ignoring the electrostatic properties of the dyes, which may cause non -specific adhesion of dye conjugates to the sample substrate. Non-specific binding is a non-trivial problem when studying targets such as EGFR, whose complete range of behaviour in the cell membrane includes periods of immobilisation when activated [9].

In this work, we have investigated the properties of 14 chemically different dyes that can be excited by one of three commonly used laser lines (491 nm, 561 nm and 638 nm) in order to ascertain their suitability for single-molecule work with live cell samples. Focusing on a single, high-performance passivating substrate, PEG-BSA nanogel, and a single protein of proven specificity, anti-EGFR Affibody, we have explored brightness, photostability and non-specific adhesion as key characteristics that influence data quality in single-molecule work.

We have previously demonstrated that PEG-based substrates, such as Linear PEG, branched or Star PEG and PEG-BSA nanogels, are able to reduce the extent of non-specific binding of fluorescent probes to glass substrates in presence and absence of cells [8]. This ability is mainly due to the thermodynamic and excluded-volume protein-repellent effects of highly hydrated PEG layers [18], [19]. PEG-BSA nanogels, unlike pure PEG layers, are also able to sustain cell growth without the need for adhesive peptide doping. This is beneficial because we have found adhesive peptides to be difficult to consistently incorporate in PEG layers at the correct density and variation in patterns of integrin binding and activation can alter the properties of the system under study [20], especially as the EGFR pathway is interconnected with integrin signalling [21], [22].

In our current study, we have assessed probe mobility by comparing the diffusion of fluorescently-conjugated anti-EGFR Affibodies [10] on the surface of T47D breast carcinoma cells and calculating instantaneous *D* coefficients, using the Alexa 488 conjugate as a reference. We have shown that the major cause of variability in measured probe mobility is the level of non-specific binding of dye conjugates to the substrate. Anti-EGFR Affibody-Alexa 488 has been demonstrated to be a specific probe for EGFR and displays a very low non-specific binding on PEG-BSA nanogel surfaces (**Fig. 1**). The measured *D* coefficients reported in the literature for EGFR vary widely, from 0.0025 to 0.28 µm^2^/s. These were measured using a wide range of techniques including fluorescence photobleaching recovery [23], [24], single particle tracking with colloidal gold [7], single molecule tracking (mainly using quantum dots) [9], [25], [26], [27], [28], fluorescence correlation spectroscopy and image correlation spectroscopy [29], [30], [31], and fluorescence intensity distribution analysis [32]. Because of the wide range of values of *D* reported, the wide range of techniques used, and the varying expression levels of the receptor, it is difficult to draw any conclusions, except to say that the mean *D* coefficient for EGFR labelled with anti-EGFR Affibody-Alexa488 measured under our experimental conditions (0.060±0.026 µm^2^/s) falls within the range previously measured. When *D* is measured only from molecules that are definitely mobile (**Fig. 4A**), we obtain an average value around 0.17 µm^2^/s, which is also within the previously measured range. The tracking data allows us to conclude that the CF640R conjugate, whose diffusion is not statistically significantly different from that of the Alexa 488 conjugate, accurately and specifically reports on the diffusion of EGFR.

The *D* coefficients measured for the other dye conjugates are lower, and in some cases very significantly lower, than the “true” value measured using Alexa 488. This is a significant effect, with some dyes showing over an order of magnitude lower *D* coefficients than that of Alexa 488. Where single molecule measurements are used to determine the diffusion coefficients of molecules, this variation in *D* raises the possibility of significant errors in diffusion coefficients calculations if the wrong dyes are used. Non-specific binding of dye molecules to the substrate would be expected to be a result of either charge-based interactions or hydrophobic interactions. It has been stated that high dye net charge, and in particular negative charge, can be responsible for non-specific binding [33] but, under our experimental conditions, we find that there is only a very weak correlation between non-specific binding and charge. Some dyes that are quite highly negatively charged show low levels of non-specific binding, and therefore high measured *D* coefficient (e.g. Alexa 488), while others with similar charge bind strongly to the substrate (e.g. Alexa 546). On the other hand, we have demonstrated a significant correlation between hydrophobicity and low measured values of *D* and therefore high levels of non-specific binding to the substrate, with the best dyes (Alexa 488, CF640R) having highly negative logD values, i.e. very low hydrophobicity.

The widely varying level of non-specific substrate attachment between the dyes we tested demonstrates that photophysical characteristics alone are insufficient to determine whether a particular dye is suitable for single molecule tracking in live cells. For example, Atto 647N is often identified as a good dye for single molecule experiments, because of its relatively high resistance to photobleaching and its potential to yield a high number of photons. However, under our experimental conditions it shows a very high level of attachment to the substrate.

One other factor that should be considered when choosing a dye for single molecule experiments is the potential for the dye to influence the properties of the labelled molecule. Alterations of probe specificity and affinity for different fluorophore conjugates are well known in the field of antibody conjugation, where it is established that excess negative charges in the dye can cause loss of specificity by altering the electrostatic parameters of the antibody [34]. The effect of spectrally equivalent but chemically different dyes on probe specificity for in vivo use has been analysed for Cy5.5- and Alexa 680-conjugated antibodies [35] and for four different Near Infra-Red fluorophore conjugates of an anti-EGFR Affibody Molecule [36]. Both papers report differential binding affinity and in-vivo Tumor-to-Background ratios, but Ogawa *et al* [35] also investigate the possible causes of this difference, concluding that the presence of multiple aromatic rings and negative charges in the structure of Cy5.5 enhances the lipophilicity of the fluorophore, possibly altering the pharmacokinetic characteristics of the antibody. Conversely, Qi *et al* [36] determine that Cy5.5 and Alexa 680 anti-EGFR Affibody conjugates display equally specific binding in vivo, while SR680 and IRDye 800 CW perform remarkably poorer, however Ogawa *et al* label a humanised full-length antibody (148 kDa) at multiple sites, while Qi *et al* label the affibody (Mw ca. 14 kDa) at a single cysteine residue. The effect of single versus multiple labelling on the net charge of the protein and the differences in protein charge and size might explain the differential behaviour of the conjugates reported in the literature.

## Conclusions

The choice of organic fluorescent dyes is large and ever-increasing, however not all of them are suited to the demanding SNR and specificity requirements of single-molecule techniques. While the effect of fluorophore labeling on antibodies is well known and single instances of fluorophore optimization for in vivo work have been published, our systematic analysis of dyes suited for the laser lines 491 nm, 561 nm and 638 nm is, to our knowledge, the first to deal with visible dyes and to investigate the suitability of conjugates by analyzing brightness, photostability and specificity at single-molecule level. Our results show that non-specific binding of dye conjugates to the substrate is a significant effect, highly variable between dyes. It is therefore important to consider this in addition to photophysical characteristics when selecting a dye. We have demonstrated that hydrophobicity is the major determinant of the propensity of a dye for binding to the substrate. We therefore suggest that hydrophilic dyes (strongly negative logD) with good photophysical characteristics should be selected in the first instance. Of the dyes we have examined, Alexa 488 appears to be the dye of choice for excitation with blue light, TMR for green, and CF640R for red. Although we have carried out our experiments on T47D cells, we believe our conclusions should be valid for a wide range of single molecule experiments, using different cell lines, as the forces modulating dye-substrate interactions will not change. We have previously published data on CHO cells, that shows higher levels of binding to the substrate for Atto 647N-affibody than for Alexa 546-affibody [8]. However, before undertaking experiments, dye conjugates should be tested under the specific conditions to be used, and their effect (or lack of it) on the labelled molecule should be investigated.

## Materials and Methods

### Surface Passivation of Glass-bottomed Dishes with PEG-BSA Nanogels

Glass-bottom cell culture dishes (35 mm dishes, 7 mm glass, No. 0 thickness, MatTek Corporation) were used for all surface treatments. PEG-BSA nanogels were prepared using 8-arm PEG-vinyl sulfone and BSA, as described by Tessler *et al* [11]. Dishes were first cleaned with piranha solution and treated with APTES as described above. 10% w/v nanogel in PBS was added to the dishes, and they were incubated for 1 hour at 37°C. The dishes were then washed in PBS and incubated for 1 hour at 37°C with 50 mg/ml BSA in PBS. The dishes were exposed to 1 M Tris, pH 8.0 for 15 minutes at room temperature to quench unreacted vinyl sulfone groups. Finally, dishes were washed with PBS. Dishes were filled with PBS to prevent layer desiccation and stored at 2–8°C for use within 2–4 weeks.

### Fluorescent Labelling of Proteins

Anti-EGFR Affibody (Abcam) was labelled at a single cysteine residue in a 1∶1 stoichiometry following the manufacturer’s instructions with the following maleimide dyes: Alexa 488, Alexa546, Alexa555, Tetramethylrhodamine-6, Bodipy FL, and Rhodamine Red C2 (Molecular Probes -Invitrogen), Cy3 (GE Healthcare), CF488A, CF568, CF633 and CF640R (Biotium), Atto565 and Atto647N (AttoTec), and Fluorescein-5 (Sigma Aldrich).

### Cell Culture

T47D cells (ECACC) were cultured in RPMI 1640 with Phenol red supplemented with 10% FCS, 2 mM L-Glutamine, 1% penicillin/streptomycin and 10 mM Sodium Pyruvate (all Invitrogen). Cells were plated on PEG-BSA nanogel-coated glass-bottomed dishes at a density of 3×10^5^ cells/dish.

Cells were rinsed twice with serum-free medium and starved for 2 hours upon reaching 80% confluence to remove serum-derived growth factors which can interfere with probe binding.

### Cell Labelling

Starved cells were rinsed twice with Serum-Free Medium pre-heated at 37°C and labelled with 4 nM each anti-EGFR Affibody for 15 minutes at 37°C. Cells were rinsed twice with SFM+HEPES 25 mM, pH 7.2 pre-heated at 37°C and promptly imaged as described below.

For bleaching assessment, starved cells were rinsed twice with chilled PBS pH 7.4 and labelled with the appropriate amount of EGFR Affibody for 1 h on ice. Cells were rinsed twice with chilled PBS pH 7.4 and fixed in 3% paraformaldehyde (Electron Microscopy Sciences), 0.5% glutaraldehyde (Sigma) for 30 minutes prior to imaging.

### Single-molecule Data Acquisition

Single- molecule images were acquired using a Zeiss Axiovert TIRF-setup with excitation wavelengths λ = 491 nm (100 mW, Cobolt Calypso), 561 nm (100 mW, Oxxius SLIM), 639 nm (30 mW, PTI IQIC30), as described previously [37]. The field of view of each channel for single-molecule imaging was 80×30 µm. Tracking data of triply labelled cells was acquired at 20 Hz for 30 seconds. At least 10 areas were acquired over three independent replicates for each experimental condition.

For bleaching assessment, data were acquired at 10 Hz for 50 seconds. At least 5 areas were acquired for each dye. The excitation power and TIRF angle was kept constant for all conjugates excited by the same laser line. Images were saved in HDF5 format for subsequent processing using custom-designed software [38].

### Analysis of Tracking Data

All single-molecule time series data were analysed using the multidimensional analysis software described in [16]. Registration transformations were determined but feature detection and tracking was performed independently in each channel. Single-molecule tracks whose mean positions fell within image regions identified as belonging to cells were selected for analysis. The Mean Square Displacement (MSD), is defined as MSD(*ΔT*) = <|*r_i_*(*T*+*ΔT*)−*r_i_*(*T*)|^2^> where |*r_i_*(*T*+*ΔT*)−*r_i_*(*T*)| is the displacement between position of track i at time T and time T+*ΔT*. A MSD curve was determined for each individual track and a straight line was fitted to the first 3 points of that MSD curve. The instantaneous diffusion coefficient, *D*, was then determined directly from the gradient, *m*, of the line as *D* = *m*/4. The mean of all the *D* values determined for a specific dye-conjugate imaged under the same conditions was used as a metric to compare the mobility of probes on the cell membrane. The mean *D* for each conjugate was also compared with the mean value of *D* determined for the anti-EGFR Affibody Alexa 488 using a two-tailed independent t-test.

### Direct Measurement by Spot Density of Dye-substrate Binding

0.5 nM of dye-conjugated anti-EGFR affibody species in Serum-Free Medium +25 mM HEPES pH 7.2 were reacted with PEG-BSA nanogel-coated dishes for 10′ at 37°C, under the same experimental conditions used for cell tracking experiments, then rinsed twice with SFM+HEPES and imaged at 37°C as described above. At least 10 independent areas were acquired for each experimental condition. Raw data was saved in HDF5 format and analysed with custom software as described above. The number of single-molecule spots for each dye channel was calculated by the analysis software and divided by the surface of the imaged area. Resulting single-molecule spot density values were logged in a spreadsheet and correlated with relevant electrostatic dye parameters (net charge and logD pH 7.4).

### Analysis of Brightness and Photobleaching Data

To determine single-molecule bleaching parameters, for every dataset, feature intensity v. time traces were extracted and all traces pertaining to the same experimental conditions were combined. A single exponential decay was fitted to the data in Origin 8 to determine the photobleaching time constant. Feature intensities of single molecules tracked within image series were combined to produce feature intensity histograms for each dye and a Gaussian or sum of Gaussians model was fitted to the data as appropriate. The position of the first peak was taken to be the mean intensity of a single molecule. The number of detected photons, 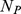, was calculated from the measured fluorescence intensities, *I*, using 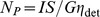, where the sensitivity of the detector, *S*, in electrons per digital level and the efficiency of the detector, 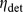, were taken from performance test data supplied by the manufacturer and specific to the EMCCD used. *G* was the EM gain setting used during image acquisition.
